# Does Male Infantile Galactocele Always Necessitate Surgical Intervention?

**DOI:** 10.7759/cureus.18001

**Published:** 2021-09-15

**Authors:** Nada E Algethami, Abdulrahman Taha, Wahaj A Altalhi, Abeer I Alsulaimani

**Affiliations:** 1 Medicine, Al Hada Armed Forces Hospital, Taif, SAU; 2 Pediatric Surgery, Raparin Teaching Hospital for Children, Erbil, IRQ; 3 Medicine, Taif University, Taif, SAU

**Keywords:** galactocele, infant, male, breast, enlargement

## Abstract

Galactocele is cystic dilatation of the mammary gland, which contains milk. Galactocele is usually presented as a painless breast enlargement with a fluctuant, soft, mobile, and non-tender mass in the breast. It is a rare disorder in the male pediatric age group, with only 31 cases reported in the literature. In this article, we present another case of unilateral galactocele in a 14-month-old male infant. Ultrasound imaging confirmed a simple cystic hypoechoic mass in the right breast, and needle aspiration was performed before surgery as a diagnostic procedure (a 1-cc extract revealed a milky-colored fluid).

## Introduction

A galactocele is a benign breast condition that is characterized by cystic dilatation of a mammary gland that contains milk [1]. Galactoceles are uncommon in the male pediatric age group, and typically, unilateral rather than bilateral cases have been identified [2,3].

The cause of galactoceles is unknown. They have been linked to three factors, i.e., previous or current prolactin stimulation, involvement of the secretory breast epithelium, and any ductal obstruction; in addition, galactoceles have been linked to certain endocrinologic disorders [4]. It is an uncommon condition. Thus far, only 31 cases have been reported. Our case of a galactocele in a male infant is the 32nd case.

## Case presentation

A 14-month-old male infant presented with an enlarged right-side breast. The enlargement was bilateral since birth; then, the left side gradually decreased in size and subsided at four months of age. However, the right side gradually increased in size and was noticed by the mother.

Upon physical examination, the patient was well and active with stable vital signs. Breast examination identified a cystic round swelling (approximately 4 × 3 cm in size) in the right breast, the nipple and areola were normal, the breast was not tender, and there were no signs of infection. There was no nipple discharge, and the genitalia were normal. There were no signs of endocrinologic abnormalities such as breast swelling obesity, feminizing adrenal cortical tumor, testicular tumor, prolactinoma, or Klinefelter syndrome.

General examination was normal regarding the height, weight, no signs of precocious puberty, and no signs of recognized syndromes. Hormonal assays showed low levels of luteinizing hormone (0.100 mIU/mL), follicle-stimulating hormone (1.29 mIU/mL), and estradiol (E2) (5.0 pg/mL). Other endocrine hormones were within the normal range; specifically, cortisol at 8 AM was 409.6 nmol/L, thyroid-stimulating hormone was 1.92 uIU/mL, total thyroxine was 69.19 µg/dL, and prolactin was 14.24 ng/mL.

**Figure 1:**
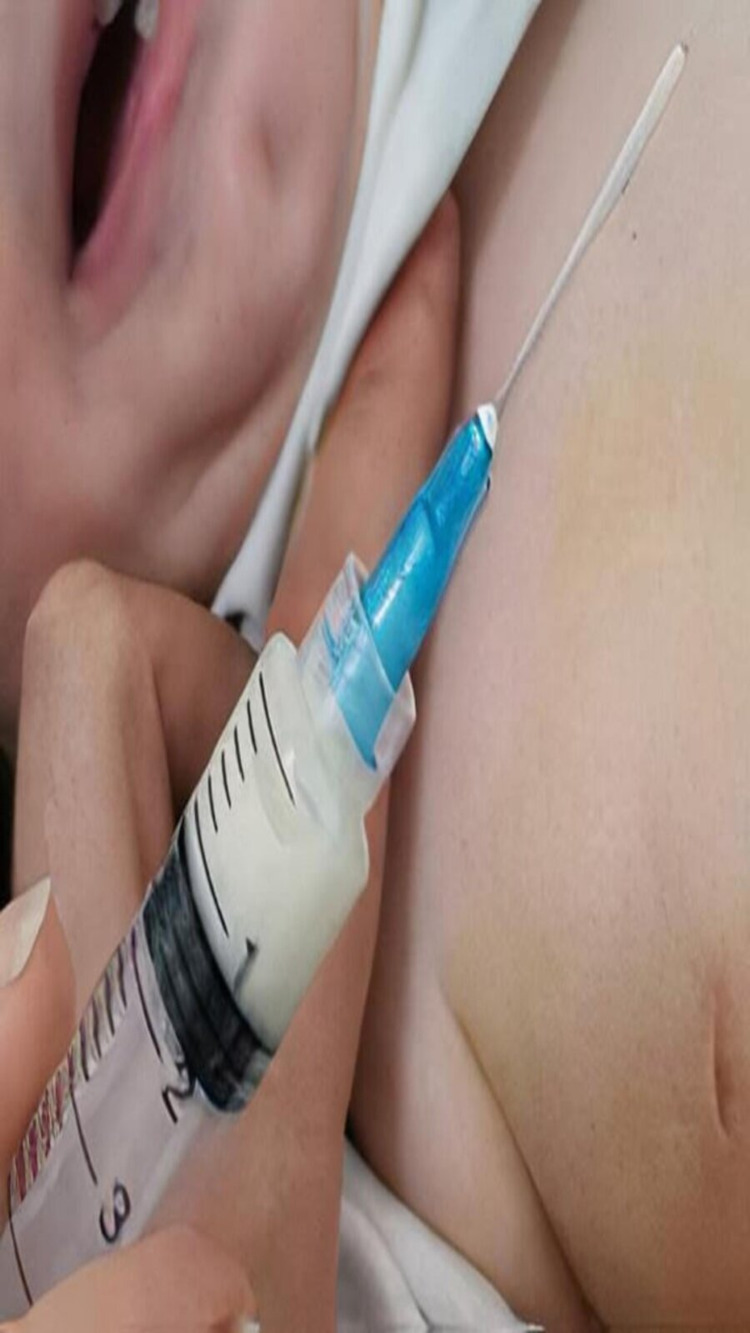
Preoperative picture of needle aspiration revealed a milky-colored fluid

**Figure 2:**
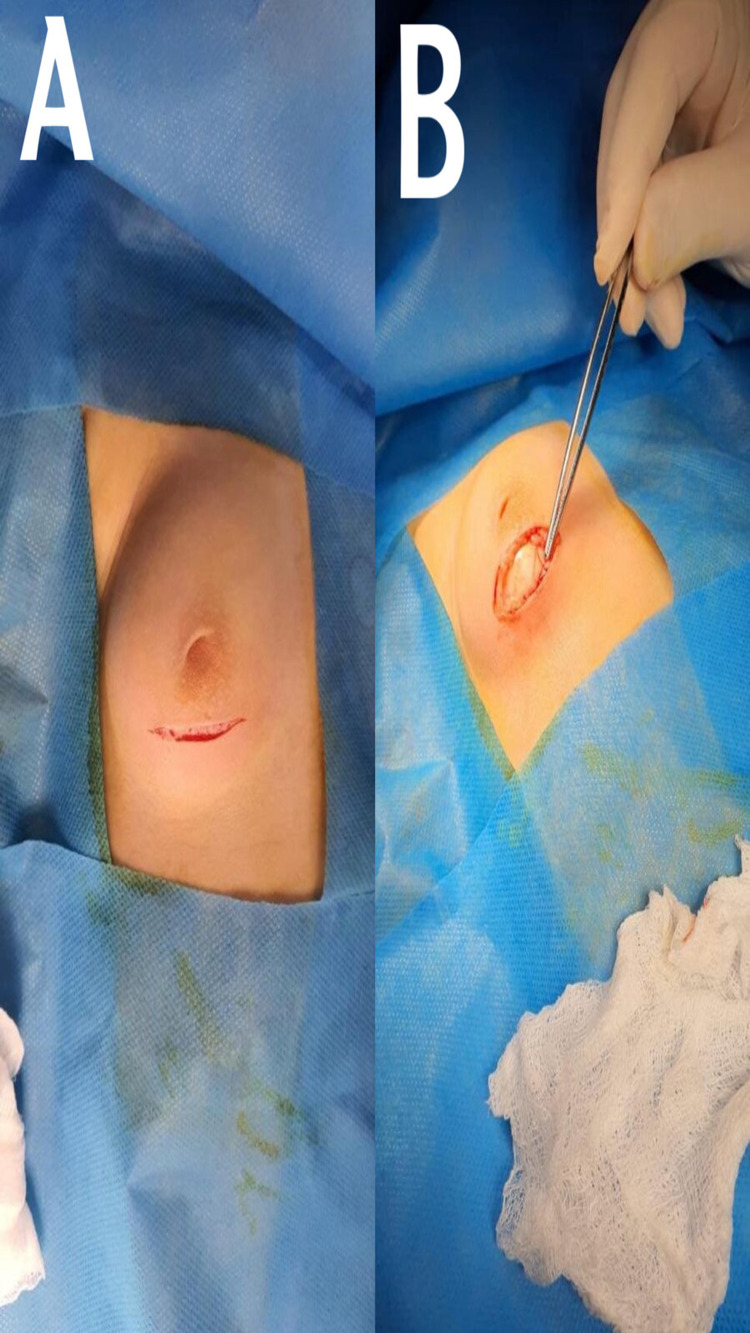
Intraoperative picture. A: Transverse subareolar incision. B: Cystic mass excision.

Ultrasound imaging confirmed a simple cystic right breast hypoechoic mass (approximately 2.6 × 3.6 cm in size), which appeared clear with echogenic material floating, but the left breast appeared normal. Needle aspiration was performed under aseptic conditions before surgery as a diagnostic procedure, and no type of anesthesia was used; a 1-cc extract revealed a milky-colored fluid, and a diagnosis of a galactocele was highly suspected (Figure 1). The cystic mass was excised by transverse subareolar incision (Figure 2A, 2B). Macroscopic histopathological examination of the resected specimen revealed a single piece of tissue measuring 20 × 15 × 10 mm, and the acute section was gray with many cystic spaces. Microscopic examination showed many cystic structures lined by cuboidal epithelium containing proteinaceous debris and foamy macrophages supported by a fibrous wall. The intervening stroma contained many land-like ductal structures. There was no atypia. The biopsy result confirmed a breast galactocele. Post-operative follow-up was done for approximately three months with no recurrence of swelling or complications, and no post-operative investigations have been done.

## Discussion

The first case of galactocele in male infants was reported in 1880 by Dr. Cattani [5]. Vlahovic et al. (2012) reported two cases of unilateral galactocele in young boys; the median age of onset of a galactocele was seven months, and clinical presentation was present at 15 months [6].

Galactoceles are usually presented in the clinic as painless breast enlargements with a fluctuant, soft, mobile, and non-tender mass in the breast, and they are typically discovered during a physical examination [4]. This observation was reported in a case of a galactocele in a two-year-old child [4]. The same was observed in our case, where the galactocele was presented as painless cystic swelling in the right breast.

Differential diagnosis of breast enlargement in infants includes galactoceles, lymphatic malformations [7], hemangioma, ductal ecstasies, and hypertrophic mastitis [2,6,8]; breast abscess is the most common differential diagnosis [6,8].

Needle aspiration of the cyst resolved the dilemma of diagnosis. Some authors consider it to be a very invasive diagnostic procedure, as revealed by a case report of a galactocele in a 15-month-old male [9]. Some authors have suggested that aspiration may eliminate the need for surgery and consider it a definitive therapeutic procedure [2-4,10].

The above-mentioned studies emphasized that pediatricians should be aware of galactoceles as a differential diagnosis of breast enlargement in infants [2-4,11]. Needle aspiration of the cyst is usually performed by surgeons during diagnostic or operative procedures [11]. In our case, we performed a diagnostic needle aspiration of the cyst; it showed a milky-colored fluid, which suggested the diagnosis of a galactocele, which was subsequently confirmed by histopathology.

Ultrasonography revealed a well-defined subcutaneous fluid collection or a complex mass [10,2]. Palpable breast masses are rare in the male pediatric patient, and sonography is the primary imaging modality [10,2]. MRI as a diagnostic procedure was performed in only four cases [10,12]. In infants, no unique mammographic findings have been observed; however, plain mammograms can show a water-fat level that is typical of galactoceles in adults [6,10].

Most have reported that surgical treatment of galactoceles by simple excision through intra-areolar incision is curative [7,9,10,13-18]. Although not all cases of male galactocele need surgical intervention, in our case, the pathology was bilateral after birth, and then, the left side gradually subsided at four months of age with no clinical or sonographic finding after one year of age. The right side became gradually larger and guaranteed surgical intervention.

## Conclusions

A breast galactocele in male infants is a rare condition, and needle aspiration can be used as a diagnostic and therapeutic modality. If needed, breast ultrasonography is the preoperative investigative technique of choice. Not all cases of male galactocele require surgical intervention because a period of conservative follow-up is acceptable if it is not symptomatic, and if needed, surgery can be performed by a simple transverse subareolar incision.
